# Biologic refractory disease in rheumatoid arthritis: results from the British Society for Rheumatology Biologics Register for Rheumatoid Arthritis

**DOI:** 10.1136/annrheumdis-2018-213378

**Published:** 2018-07-06

**Authors:** Lianne Kearsley-Fleet, Rebecca Davies, Diederik De Cock, Kath D Watson, Mark Lunt, Maya H Buch, John D Isaacs, Kimme L Hyrich

**Affiliations:** 1 Arthritis Research UK Centre for Epidemiology, Manchester Academic Health Science Centre, The University of Manchester, Manchester, UK; 2 Leeds Institute of Rheumatic and Musculoskeletal Medicine, University of Leeds, Leeds, UK; 3 National Institute of Health Research Leeds Biomedical Research Centre, Leeds Teaching Hospitals NHS Trust, Leeds, UK; 4 Institute of Cellular Medicine, Newcastle University and National Institute for Health Research Newcastle Biomedical Research Centre, Newcastle upon Tyne Hospitals NHS Foundation Trust, Newcastle upon Tyne, UK; 5 National Institute of Health Research Manchester Biomedical Research Centre, Manchester Academic Health Science Centre, Manchester University NHS Foundation Trust, Manchester, UK

**Keywords:** rheumatoid arthritis, epidemiology, dmards (biologic)

## Abstract

**Objectives:**

Biologic disease-modifying antirheumatic drugs (bDMARDs) have revolutionised treatment and outcomes for rheumatoid arthritis (RA). The expanding repertoire allows the option of switching bDMARD if current treatment is not effective. For some patients, even after switching, disease control remains elusive. This analysis aims to quantify the frequency of, and identify factors associated with, bDMARD refractory disease.

**Methods:**

Patients with RA starting first-line tumour necrosis factor inhibitor in the British Society for Rheumatology Biologics Register for RA from 2001 to 2014 were included. We defined patients as bDMARD refractory on the date they started their third class of bDMARD. Follow-up was censored at last follow-up date, 30 November 2016, or death, whichever came first. Switching patterns and stop reasons of bDMARDs were investigated. Cox regression identified baseline clinical factors associated with refractory disease. Multiple imputation of missing baseline data was used.

**Results:**

867 of 13 502 (6%) patients were bDMARD refractory; median time to third bDMARD class of 8 years. In the multivariable analysis, baseline factors associated with bDMARD refractory disease included patients registered more recently, women, younger age, shorter disease duration, higher patient global assessment, higher Health Assessment Questionnaire score, current smokers, obesity and greater social deprivation.

**Conclusions:**

This first national study has identified the frequency of bDMARD refractory disease to be at least 6% of patients who have ever received bDMARDs. As the choice of bDMARDs increases, patients are cycling through bDMARDs quicker. The aetiopathogenesis of bDMARD refractory disease requires further investigation. Focusing resources, such as nursing support, on these patients may help them achieve more stable, controlled disease.

## Introduction

Biologic disease-modifying antirheumatic drugs (bDMARDs) have revolutionised treatment pathways for rheumatoid arthritis (RA) management, improving outcomes for patients who do not tolerate or respond to conventional synthetic (cs)DMARDs. However, for some patients, even after multiple bDMARDs, disease control is unachievable with so-called ‘difficult-to-treat’^1^ or bDMARD refractory disease.^2^

The repertoire of bDMARDs is continually expanding. Tumour necrosis factor inhibitors (TNFi) remain the first-line bDMARD for patients with RA.^3^ There are additional cytokine-targeted therapies licensed for RA, including interleukin (IL)-6 pathway inhibitors, IL-1 receptor antagonists, cell-targeted B-cell depleting agents and T-cell costimulation blockers. Some patients may fail their bDMARD due to ineffectiveness, either true lack of effect or non-adherence, adverse effects or intolerance. The National Institute for Health and Care Excellence (NICE) have published guidance recommending rituximab in patients who have failed at least one TNFi unless contraindicated.^4^ With increasing treatment options, patients may cycle through several bDMARDs, although the precise extent to which this occurs in clinical practice is unknown. As further bDMARDs are introduced for RA, it also challenges the definition of bDMARD refractory disease, both in clinical and research settings. This is an important area of investigation as there are no current guidelines on optimal bDMARD sequencing beyond a second bDMARD.^4^

The British Society for Rheumatology Biologics Register for RA (BSRBR-RA) is a national ongoing treatment register, capturing bDMARD exposures, treatment response and adverse effects across a large population of patients with RA from the UK. This unique setting may improve understanding of bDMARD refractory disease. The specific analysis objectives were to (1) quantify what proportion of patients starting their first TNFi will subsequently exhibit bDMARD refractory disease, (2) describe bDMARD treatment patterns over time and reasons for sequential use in these patients, and (3) identify clinical predictors of bDMARD refractory disease early in the bDMARD treatment pathway.

## Methods

### Study setting

The BSRBR-RA, established in 2001, is a national prospective observational cohort study. It collects data of adults with a physician’s diagnosis of RA starting a bDMARD. The overall aim of the register is to monitor long-term safety of bDMARDs in the clinical setting. At start of therapy, baseline data are collected including demographics (age, gender, height, weight, smoking status, comorbidities), disease characteristics (disease duration, rheumatoid factor (RF) status, joint erosions on X-ray), disease activity (swollen and tender joint count, patient global assessment, erythrocyte sedimentation rate (ESR) and/or C reactive protein) and 28-joint disease activity score (DAS28),^5^ Health Assessment Questionnaire (HAQ)^6^ for patient function, Medical Outcomes Study 36-item short form health survey (SF-36),^7^ and current or previous antirheumatic therapies. Follow-up data on disease activity, disease function and antirheumatic therapies are collected every 6 months for 3 years, with disease activity and antirheumatic therapy data collected annually thereafter. Full details of the BSRBR-RA methodology have been published previously.^8^ Ethics approval for the BSRBR-RA was granted by the North West Multicentre Research Ethics Committee in December 2000 (MREC 00/8/53). No additional ethical approval was required for the current analysis.

### Exposure to bDMARDs

Patients starting TNFi were recruited from 2001 to 2008, and again from 2011 onwards. This analysis included all patients starting a TNFi as their first bDMARD between 1 October 2001 (study start) until 30 November 2014 (2 years prior to analysis cut-off date to allow sufficient follow-up). NICE allow bDMARD treatment for patients with RA with DAS28 >5.1 despite treatment with at least two csDMARDs.^3^ For each patient, the total number of bDMARD treatment courses was identified, irrespective of bDMARD class or whether the bDMARD had been received previously. Subsequently, for each patient, all treatment courses were reviewed and clustered according to bDMARD class: TNFi (adalimumab, certolizumab, etanercept, golimumab, infliximab), B-cell-depleting agent (rituximab or ocrelizumab), IL-1 receptor antagonist (anakinra), IL-6 pathway inhibitor (tocilizumab) and T-cell costimulation blocker (abatacept). Patients who had been exposed to at least three different classes of bDMARD (irrespective of reason for failure to prior bDMARD) were classified as ‘bDMARD refractory’. The number of bDMARDs that patients were exposed to, as well as the number of bDMARD classes, are presented in online supplementary table 1.

10.1136/annrheumdis-2018-213378.supp1 Supplementary data

### Statistical analysis

Follow-up started on the date of first TNFi exposure. Patients were defined as bDMARD refractory on the date they started their third class of bDMARD. Patients were censored at their last follow-up date, 30 November 2016 (analysis cut-off), or date of death, whichever came first. Switching patterns and reasons for stopping bDMARDs were presented for all bDMARD refractory patients. Kaplan-Meier analysis was used to quantify bDMARD refractory disease. Body mass index (BMI) was calculated for each patient. Data outside the BMI range of 14 to 50 were assumed incorrect. Obesity was classified if BMI was 30 or greater.^9^ Index of multiple deprivation (IMD) quintiles were calculated for England,^10^ Scotland^11^ and Wales^12^ separately, then combined into an overall IMD quintile score. Quintile scores for Northern Ireland were unavailable. Cox regression analysis was used to identify baseline clinical factors associated with bDMARD refractory status. Results were presented as HRs with 95% CI. The SF-36 physical component score was excluded from the multivariable analysis due to the strong association with HAQ (correlation 0.6). A sensitivity analysis including patients recruited from 2011 onwards was completed.

### Multiple imputation

Multiple imputation (49 iterations based on proportion of incomplete cases^13^) was used to account for missing baseline covariate data. Complete variables included bDMARD refractory status, registration year, registered TNFi, gender, age at first TNFi, comorbidities and follow-up time until failure or end of study. Imputed values included disease duration at start of first TNFi, tender joint count, swollen joint count, physician global assessment, ESR, DAS28, HAQ, SF-36 physical component score, SF-36 mental component score, RF status, erosions on X-ray, smoking status and BMI. Stata V.13 was used to perform all analyses.^14^

## Results

### Baseline characteristics

A total of 13 502 patients were registered at start of first TNFi between 2001 and 2014 (table 1), the majority recruited in the first 8 years (86%); 76% women, median age 57 years (IQR 49–65), median disease duration 10 years (IQR 5–18). Disease activity and severity at the start of first TNFi was high; median DAS28 6.5 (IQR 5.8–7.2), median HAQ 2.0 (IQR 1.6–2.4). Over half (53%) reported at least one comorbidity at start of first TNFi, and 22% were current smokers.

**Table 1:** Baseline characteristics of all 13 502 patients in the BSRBR-RA starting a first-line TNFi between 2001 and 2014

	All patients	bDMARD refractory	Remaining patients
N	13 502	867	12 635
First TNFi (n=13 502)			
Etanercept	4612 (34%)	285 (33%)	4327 (34%)
Infliximab	3794 (28%)	246 (28%)	3548 (28%)
Adalimumab	4322 (32%)	391 (34%)	4031 (32%)
Certolizumab	774 (6%)	45 (5%)	729 (6%)
Registration year (category) (n=13 502)	–	–	–
2001–2008	11 654 (86%)	778 (90%)	10 876 (86%)
2011–2014	1848 (14%)	89 (10%)	1759 (14%)
Women (n=13 502)	10 269 (76%)	705 (81%)	9564 (76%)
Age (years) (n=13 502)	57 (49 to 65)	52 (44 to 59)	58 (49 to 66)
Age (category) (n=13 502)	–	–	–
16–50	3888 (29%)	381 (44%)	3507 (28%)
51–90	9614 (71%)	486 (56%)	9128 (72%)
Disease duration (years) (n=13 360)	10 (5 to 18)	9 (4 to 16)	10 (5 to 18)
Disease duration (category) (n=13 360)	–	–	–
0–10	6835 (51%)	494 (57%)	6341 (51%)
11–72	6514 (49%)	368 (43%)	6157 (49%)
Concurrent methotrexate (n=13 502)	8537 (63%)	578 (67%)	7959 (63%)
Concurrent steroids (n=13 502)	5620 (42%)	364 (42%)	5256 (42%)
Total comorbidities† (n=13 502)	–	–	–
None	6327 (47%)	408 (47%)	5919 (47%)
1 comorbidity	4589 (34%)	294 (34%)	4295 (34%)
2 comorbidities	1894 (14%)	122 (14%)	1772 (14%)
3+ comorbidities	692 (5%)	43 (5%)	649 (5%)
Smoking status (n=13 351)	–	–	–
Current smoker	2899 (22%)	248 (29%)	2651 (21%)
Ex-smoker	5068 (38%)	284 (33%)	4784 (38%)
Never smoked	5384 (40%)	330 (38%)	5054 (40%)
Body mass index (kg/m^2^) (n=11 499*)	26 (23 to 30)	26 (23 to 31)	26 (23 to 30)
Obese (body mass index ≥30) (n=11 499*)	2951 (26%)	224 (30%)	2727 (25%)
Disease activity	–	–	–
Tender joint count (range 0–28) (n=13 091)	15 (10 to 22)	16 (11 to 23)	15 (10 to 21)
Swollen joint count (range 0–28) (n=13 083)	10 (6 to 15)	11 (7 to 16)	10 (6 to 15)
Patient global assessment (range 0–10 cm) (n=13 000)	7.5 (6.2 to 8.7)	7.8 (6.6 to 9.0)	7.5 (6.1 to 8.6)
ESR (mm/s) (n=12 084*)	38 (22 to 62)	36 (22 to 60)	38 (22 to 62)
CRP (mm/s) (n=5274*)	27 (12 to 57)	28 (11 to 56)	27 (12 to 57)
DAS28 (range 0–10) (n=13 255)	6.5 (5.8 to 7.2)	6.6 (5.9 to 7.3)	6.5 (5.8 to 7.2)
HAQ (range 0–3) (n=12 364*)	2.0 (1.6 to 2.4)	2.1 (1.8 to 2.5)	2.0 (1.6 to 2.4)
SF-36: Physical Component Score‡ (n=8702*)	15 (10 to 21)	14 (10 to 19)	15 (10 to 21)
SF-36: Mental Component Score‡ (n=8702*)	42 (34 to 51)	40 (32 to 50)	42 (34 to 51)
Index of multiple deprivation (excluding Northern Ireland) (n=12 711*)	–	–	–
Lowest quintile (most deprived)	2082 (16%)	165 (20%)	1917 (16%)
Middle 3 quintiles	8008 (63%)	494 (61%)	7514 (63%)
Highest quintile (least deprived)	2621 (21%)	147 (18%)	2474 (21%)

Results presented as N (%) or median (IQR).

*More than 5% missing data.

†Total comorbidities—hypertension, ischaemic heart disease, stroke, lung disease, renal disease, diabetes, depression, liver disease. ‡SF-36; greater score indicates better health.

bDMARD, biologic disease-modifying antirheumatic drug; BSRBR-RA, British Society for Rheumatology Biologics Register for rheumatoid arthritis; CRP, C reactive protein; DAS28, 28-joint Disease Activity Score; ESR, erythrocyte sedimentation rate; HAQ, Health Assessment Questionnaire; SF-36, 36-item Short Form Survey for quality of life; TNFi, tumour necrosis factor-alpha inhibitor.

### bDMARD refractory patients

**Figure 1:**
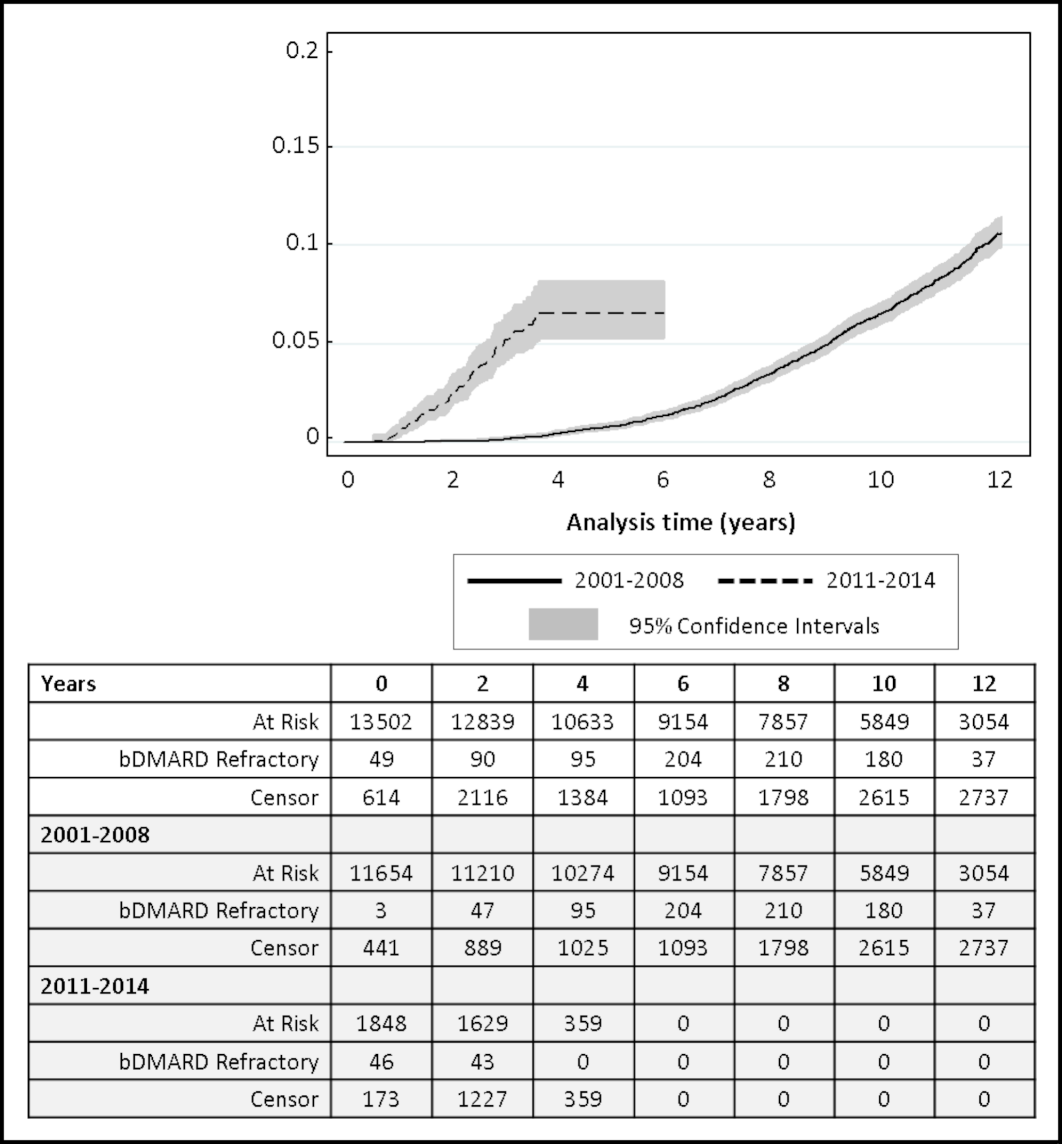
Cumulative incidence plot (95% CIs) of when patients acquire biologic disease-modifying antirheumatic drug (bDMARD) refractory disease (point of starting their third class of bDMARD) (n=13 502). Stratified by recruitment year: prior to 2001–2008 (solid line; n=11 654) and 2011–2014 (dashed line; n=1848).

Over 111 034 person-years of follow-up, 867 (6.4%) patients were classified as bDMARD refractory (exposed to at least three different classes of bDMARD); median time from first TNFi to bDMARD refractory disease 7.9 years (95% CI 5.7 to 10.0) (figure 1). A higher proportion (6.7%) of patients from the earlier recruitment cohort (2001–2008) had bDMARD refractory disease with a longer median time of 8.4 years (95% CI 6.6 to 10.2) to refractory status. In contrast, 4.8% of patients in the 2011–2014 cohort were bDMARD refractory over a shorter median time of 2.0 years (95% CI 1.4 to 2.6). Overall, patients with bDMARD refractory disease remained on their first TNFi for a median of 3.9 years (IQR 1.5–6.6); longer in the earlier recruitment cohort (4.4 years vs 0.8 years, respectively). Reasons for patients stopping their first TNFi were 452 (52%) for ineffectiveness, 205 (24%) following adverse events, 29 (3%) for other reasons (mostly patient choice due to injection-related problems or family planning) and 181 (21%) not recorded. Stop reasons were similar between the recruitment cohorts. Overall, 331 (38%) reported repeated ineffectiveness, 95 (11%) reported repeated adverse events, 383 (44%) reported a mixture of stop reasons, while 58 (7%) had missing stop reasons. Patients with bDMARD refractory disease then spent a median of 1.5 years on their second class (IQR 0.8–2.6) and 1.5 years on their third class of bDMARD (IQR 0.8–2.8), although this was longer in patients recruited 2001–2008 compared with 2011 onwards; 1.5 versus 0.8 years, and 1.6 versus 1.0 years for second and third bDMARD class, respectively. Overall, 5% of the bDMARD refractory patients died over follow-up, lower compared with the remaining population (11%).

### bDMARD treatment pathways

**Figure 2:**
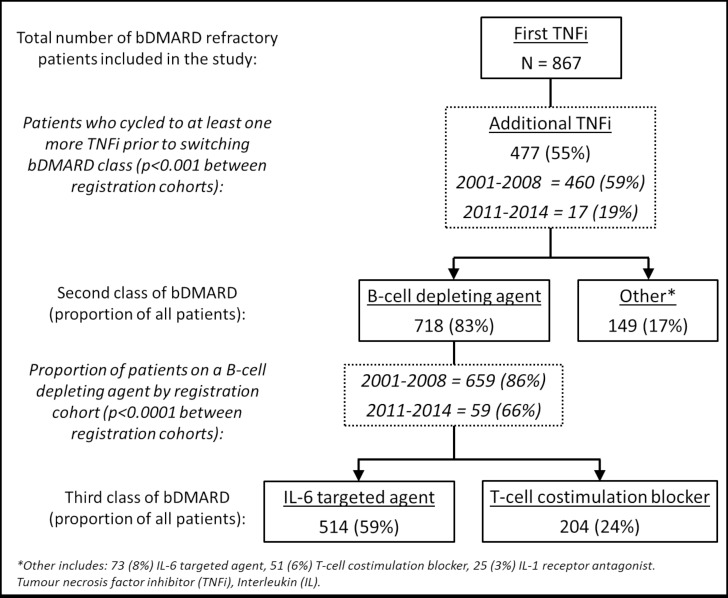
Main pattern of biologic disease-modifying antirheumatic drug (bDMARD) class switching in the 867 bDMARD refractory patients.

The majority of bDMARD refractory patients switched to a B-cell-depleting agent as their second class of bDMARD (n=718; 83%), although the proportion reduced after 2011 (66% vs 85%; p<0.001) (figure 2). The two most common class-switching pathways was from TNFi to B-cell-targeted agent rituximab (aside from use of ocrelizumab in two patients) to either IL-6-targeted agent tocilizumab (n=514; 59%) or T-cell costimulation blocker abatacept (n=204; 24%). Many bDMARD refractory patients had been exposed to multiple bDMARDs within each class. More patients recruited in the earlier years had received at least one more TNFi before switching to their second class of bDMARD (59% vs 19%; p<0.001). Most bDMARD refractory patients reported use of four different bDMARDs (n=328; 38%), 173 (20%) used five and 72 (8%) reported use of at least six different bDMARDs. Twenty per cent of the bDMARD refractory patients reported more than three classes of bDMARDs.

### Factors associated with bDMARD refractory disease

In the multivariable analysis (table 2), bDMARD refractory disease was associated with women (HR 1.3; 95% CI 1.1 to 1.5), younger age (HR 0.6 for age >50 years; 95% CI 0.5 to 0.7), shorter disease duration (HR 0.8 for disease duration >10 years; 95% CI 0.7 to 0.95), higher patient global assessment (HR 1.1 per cm; 95% CI 1.0 to 1.1), higher HAQ (HR 1.3 per unit; 95% CI 1.1 to 1.5), current smoking (HR vs never 1.5; 95% CI 1.2 to 1.7) and obesity (HR 1.2 for BMI ≥30; 95% CI 1.0 to 1.4) at the start of first TNFi. Notably, the HR for developing bDMARD refractory disease was 15 times higher (95% CI 10 to 21) among patients recruited from 2011 onwards compared with 2001–2008. A further subanalysis that included social deprivation scores for England, Scotland and Wales also identified that patients in the lowest IMD quintile, representing the highest level of deprivation, were associated with bDMARD refractory disease (HR compared with all remaining patients 1.2; 95% CI 1.0 to 1.4). A sensitivity analysis of the 1848 patients in the 2011 to 2014 recruitment cohort supported the findings that shorter disease duration and worse HAQ were associated with bDMARD refractory RA (see online supplementary table 2).

**Table 2:** Univariable and multivariable analysis (imputed data, 49 datasets)—HRs for acquiring bDMARD refractory disease

	Univariable HR	Multivariable HR	Multivariable HR (including IMD)*
Registration year (2011–2014 vs 2001–2008)	15 (11 to 20); p<0.001	15 (10 to 21); p<0.001	17 (11 to 24); p<0.001
Women (vs men)	1.3 (1.1 to 1.5); p=0.004	1.3 (1.1 to 1.5); p=0.009	1.2 (1.0 to 1.5); p=0.04
Age, years (>50 vs ≤50)	0.6 (0.5 to 0.7); p<0.001	0.6 (0.5 to 0.7); p<0.001	0.6 (0.5 to 0.7); p<0.001
Disease duration, years (>10 vs ≤10)	0.7 (0.6 to 0.8); p<0.001	0.8 (0.7 to 0.95); p=0.008	0.8 (0.7 to 0.9); p=0.004
RF positive (vs negative)	1.1 (0.9 to 1.3); p=0.3	1.1 (1.0 to 1.3); p=0.1	1.1 (0.9 to 1.3); p=0.2
Erosions on X-ray (vs negative)	0.8 (0.7 to 1.0); p=0.01	1.0 (0.8 to 1.1); p=0.8	1.0 (0.8 to 1.1); p=0.7
Methotrexate at registration (vs none)	1.1 (0.9 to 1.2); p=0.3	1.0 (0.9 to 1.2); p=0.6	1.0 (0.9 to 1.2); p=0.9
On steroids at registration (vs none)	1.0 (0.8 to 1.1); p=0.6	1.0 (0.9 to 1.2); p=0.5	1.1 (0.9 to 1.2); p=0.4
Tender joint count (per joint)	1.02 (1.01 to 1.02); p=0.001	1.0 (1.0 to 1.0); p=0.3	1.0 (1.0 to 1.0); p=0.3
Swollen joint count (per joint)	1.0 (1.0 to 1.0); p=1.0	1.0 (1.0 to 1.0); p=0.9	1.0 (1.0 to 1.0); p=1.0
Patient global assessment (cm)	1.1 (1.1 to 1.1); p<0.001	1.1 (1.0 to 1.1); p=0.007	1.1 (1.0 to 1.1); p=0.02
ESR (mm/h)	0.996 (0.994 to 0.999); p=0.003	1.0 (1.0 to 1.0); p=0.6	1.0 (1.0 to 1.0); p=0.3
DAS28 (whole unit)	1.1 (1.0 to 1.1); p=0.1	1.0 (0.8 to 1.2); p=0.8	1.0 (0.8 to 1.3); p=0.8
HAQ (whole unit)	1.2 (1.1 to 1.3); p=0.005	1.3 (1.1 to 1.5); p=0.001	1.2 (1.1 to 1.4); p=0.003
Total comorbidities† (vs none)			
1 comorbidity	1.0 (0.9 to 1.2); p=0.6	1.1 (0.9 to 1.3); p=0.3	1.1 (0.9 to 1.3); p=0.2
2 comorbidities	1.2 (0.9 to 1.4); p=0.2	1.2 (0.9 to 1.4); p=0.2	1.1 (0.9 to 1.4); p=0.2
3+ comorbidities	1.3 (1.0 to 1.8); p=0.09	1.3 (0.9 to 1.8); p=0.1	1.2 (0.9 to 1.7); p=0.3
Smoke status (vs never smoked)			
Current smoker	1.5 (1.3 to 1.7); p<0.001	1.5 (1.2 to 1.7); p<0.001	1.4 (1.2 to 1.7); p<0.001
Ex-smoker	1.0 (0.8 to 1.1); p=0.7	1.1 (0.9 to 1.3); p=0.4	1.0 (0.8 to 1.2); p=1.0
Obese (body mass index ≥30)	1.3 (1.1 to 1.5); p=0.001	1.2 (1.0 to 1.4); p=0.047	1.2 (1.0 to 1.4); p=0.04
SF-36: Physical Component Score	0.97 (0.95 to 0.99); p=0.01	–	–
SF-36: Mental Component Score	0.98 (0.96 to 1.0); p=0.1	1.0 (1.0 to 1.0); p=0.4	1.0 (1.0 to 1.0); p=0.5
IMD (excluding Northern Ireland) (all other patients as referent)			
Lowest quintile (more deprived)	1.4 (1.2 to 1.7); p<0.001	–	1.2 (1.0 to 1.4); p=0.03

Results are presented as HRs with 95% CIs.

*Patients with IMD data, excluding Northern Ireland (n=12 711).

†Total comorbidities—hypertension, ischaemic heart disease, stroke, lung disease, renal disease, diabetes, depression, liver disease.

bDMARD, biologic disease-modifying antirheumatic drug; DAS28, 28-joint Disease Activity Score (higher score indicates worse health); ESR, erythrocyte sedimentation rate; HAQ, Health Assessment Questionnaire (higher score indicates worse health); IMD, index of multiple deprivation; RF, rheumatoid factor; SF-36, 36-item Short Form Survey for quality of life (higher score indicates improved health); TNFi, tumour necrosis factor-alpha inhibitor.

## Discussion

This is the first observational study to evaluate the extent of bDMARD refractory RA, defined as exposed to at least three different classes of bDMARD. Approximately 6% of patients who started TNFi as their first bDMARD were subsequently classified as bDMARD refractory. This important observation provides information that rheumatologists can use to encourage healthcare providers to address refractory patients. Quantifying the frequency of multiple bDMARD class failure is crucial, particularly in an environment where bDMARD choice is largely based on custom and experience rather than by individual biomarkers. As response to subsequent bDMARDs is known to reduce,^15 16^ targeted personalised pathways are important to identify. This knowledge can therefore drive clinical guideline development as well as inform cost-effectiveness analyses.

Prior research has suggested that patients achieve a better clinical response when switching from a first-line TNFi to rituximab compared with a second TNFi.^17^ However, many patients in the current study were recruited between 2001 and 2008 when other classes of bDMARDs were not readily available (NICE published guidance on the use of rituximab for RA in August 2007^18^ and abatacept in April 2008^19^), resulting in TNFi cycling in 59% of patients compared with only 19% in patients recruited from 2011 onwards. The majority of patients switched from TNFi to rituximab, then to either tocilizumab or abatacept, largely reflecting the order in which these drugs became available. In more recent years, there appears to have been a move away from rituximab as a second-line bDMARD class (66% compared with 85% in the earlier cohort), although our study was not designed to explore the reasons for temporal trends. Further work comparing effectiveness across different second-line therapies in large datasets is warranted.

As would be expected, the patients recruited earlier within the study (2001–2008) with a longer follow-up were more likely to be classified with bDMARD refractory disease compared with those recruited more recently (2011–2014) with shorter follow-up (6.7% vs 4.8%). However, the patients recruited more recently were 15 times more likely to have bDMARD refractory disease compared with those recruited in the earlier years. This may indicate that if these more recent patients were followed up to the same degree, the proportion classified as bDMARD refractory will likely increase over time. The explanation is likely due to increased class availability and higher expectations of bDMARDs in the more recent cohort, a result of selection bias rather than a true biologic effect. The multivariable analysis aimed to take this temporal change into account. Consequently, the burden of bDMARD refractory disease in patients recruited in the earlier years of this study are very likely an underestimate.

This analysis identified that patients from lower socioeconomic areas were more likely to develop bDMARD refractory disease. It has been previously reported that people from more deprived areas are more likely to smoke and have a higher BMI,^20^ known factors associated with drug adverse events and ineffectiveness.^1^ All three variables were found to be independent predictors of bDMARD refractory disease in our analysis. Smoking has been previously reported in association with poorer clinical response to TNFi,^21^ perhaps due to an association with high concentrations of proinflammatory cytokines.^22 23^ The proinflammatory environment associated with adipose tissue may similarly cause obese patients to respond less favourably to treatment compared with patients with a normal BMI.^1^ It may also influence the exposure to bDMARDs, especially those with a fixed dose regimen. The basis for social deprivation and association with a higher rate of bDMARD refractory disease is not immediately obvious, but may relate to other unmeasured factors such as comorbidities and/or poor adherence. Poor adherence is strongly associated with lesser DAS28 responses to bDMARDs.^24^ Unfortunately, the BSRBR-RA does not currently capture a measure of adherence. Some of the study patients may be refractory due to non-adherence. However, identifying these patients remains important regardless of the reasons for being bDMARD refractory.

Additional factors independently associated with bDMARD refractory disease were female gender, younger age, shorter disease duration, poorer patient global assessment and worse physical function. It has been reported previously that men are more likely to achieve DAS28 remission on bDMARDs.^21 25 26^ Younger patients were perhaps treated more aggressively leading to increased switching between bDMARD classes, with more caution practised in the treatment of older patients. The association between shorter disease duration and higher probability of bDMARD refractory disease is interesting, and may reflect current practice of early DMARD introduction and treatment to target, to improve outcomes.^27 28^ Function in established RA largely reflects joint damage, which is not reversed with current bDMARDs; it also associates with a poorer patient global assessment. This may drive persistently high DAS28 (in the absence of clear signs of inflammation), and thus bDMARD class switching and refractory disease status. Additionally, in the sensitivity analysis of the more recent recruitment cohort, despite reduced power, both disease duration and HAQ remained significant findings.

This analysis was set within one of the largest cohorts of patients with RA starting bDMARDs in the world. However, this analysis should be repeated in other countries where access to bDMARD drugs may differ.^29^ Patients starting a non-TNFi as first bDMARD were excluded from this analysis as use is low within the BSRBR-RA and is often due to contraindications to TNFi, providing a less representative patient population. One of the main limitations of this study is that sufficient follow-up time is needed for bDMARD refractory disease to reveal itself and, by definition, multiple bDMARDs must be available for patients to try. The survival method used to calculate the proportion of patients with refractory disease accounted for these variable follow-up times. However, it cannot account for the fact that patients may have died prior to the availability of a second or third class of bDMARD and therefore our calculation of the proportion of patients with bDMARD refractory disease is likely a minimum estimate. Hence, mortality seems lower in bDMARD refractory patients. In addition, these patients could have lower mortality because they are seen more frequently in clinic, thus significant health problems may be identified and treated earlier.

There is currently no accepted definition of bDMARD refractory RA. We defined bDMARD refractory disease as exposure to at least three different classes of bDMARDs to differentiate them from bDMARD non-responders to a single class of drug. As different bDMARDs target different components of the immune system, it may be that disease activity is driven by different pathways between individual patients. Therefore, non-response to a single bDMARD class may not represent true bDMARD refractory disease. While other definitions were considered, development of a specific definition of bDMARD refractory RA was not the remit of this specific analysis. Defining ‘difficult-to-treat’ RA is one of the current aims of a recently convened European League Against Rheumatism task force.^30^ We did not consider only those stopping for ineffectiveness in our analysis, which may be a limitation, but as an initial analysis of this important topic we elected to include all patients in order to describe the full burden of patients requiring multiple bDMARDs. In addition, it was not possible to confirm the response to treatment in those who stopped for adverse events due to missing data. The BSRBR-RA does not capture serological samples, and therefore no measures of drug levels or antidrug antibodies were possible to further delineate reasons for ineffectiveness of therapies. Finally, with the continued introduction of newer targeted (including biologic) DMARD therapies, such as the new kinase inhibitors, it also challenges the definition of when a patient should be classified as being truly bDMARD refractory.

In conclusion, this study has estimated that approximately 6% of patients who start a first-line TNFi will experience bDMARD refractory disease. This is possibly an underestimate as it excludes patients who died, and who persisted with initial therap(ies) or did not start subsequent therapies for reasons such as comorbidity. Overall, our analysis supports recent recommendations for difficult-to-treat patients with RA where evaluation of modifiable lifestyle factors such as obesity and smoking are important.^1^ Continued study of these patients is essential, particularly due to the lack of data available from randomised controlled trials of optimal treatment strategies.^31^ A better understanding of bDMARD refractory disease should help to better target expensive therapies to those patients who are most likely to respond, developing hand in hand with stratified and personalised medicine approaches.
