# Supplementary material for: Biologic refractory disease in rheumatoid arthritis: results from the British Society for Rheumatology Biologics Register for Rheumatoid Arthritis
Source: Ann Rheum Dis. 2018 Jul 6;77(10):1405–12. doi: 10.1136/annrheumdis-2018-213378 (PMC6161665; doi:10.1136/annrheumdis-2018-213378)
Supplement: Supplementary data [file annrheumdis-2018-213378supp001.docx]

**Online Supplementary Table 1: Number of patients exposed to different bDMARDs, including the number bDMARD classes patients are exposed to.**

| **Number of bDMARDs^#^** | **Number of bDMARD classes** | | | | | **Total** |
| --- | --- | --- | --- | --- | --- | --- |
|  | **1** | **2** | **3** | **4** | **5** |  |
| **1** | 7005 | - | - | - | - | **7005** |
| **2** | 2164 | 1536 | - | - | - | **3700** |
| **3** | 359 | 1139 | 247 | - | - | **1745** |
| **4** | 55 | 311 | 291 | 37 | - | **694** |
| **5** | 2 | 54 | 112 | 63 | 0 | **231** |
| **6** | 0 | 6 | 33 | 46 | 0 | **85** |
| **7+** | 0 | 4 | 12 | 24 | 2 | **42** |
| **Total** | **9585** | **3050** | **695** | **170** | **2** | **13502** |
|  | | | | | | |
| **Summary of Exposure:** | | **N (%)** | | | | |
| ≥3 bDMARDs | | 2887 (21%) | | | | |
| ≥4 bDMARDs | | 1142 (8.5%) | | | | |
| ≥5 bDMARDs | | 448 (3.3%) | | | | |
| ≥2 classes of bDMARD | | 3917 (29%) | | | | |
| ≥3 classes of bDMARD* | | 867 (6.4%) | | | | |
| ≥4 classes of bDMARD | | 172 (1.3%) | | | | |

*^#^Number of bDMARDs was assessed irrespective of bDMARD class or whether the bDMARD had been received previously. Patients with exposure to more than seven bDMARDs have been grouped together to avoid making them identifiable. *Definition used in final analysis. Biologic Disease Modifying Anti-Rheumatic Drugs (bDMARD).*

**Online Supplementary Table 2: Univariable and multivariable analysis of the 1848 patients in the 2011-14 cohort, of which 89 (4.8%) were bDMARD refractory (imputed data, 49 datasets) – hazard ratios for acquiring bDMARD refractory disease.**

|  | **Univariable Hazard Ratio** | **Multivariable Hazard Ratio** | **Multivariable Hazard Ratio (including IMD)^##^** |
| --- | --- | --- | --- |
| **Females** (vs males) | 1.0 (0.6, 1.6); p=1.0 | 1.0 (0.6, 1.6); p=0.9 | 0.9 (0.6, 1.6); p=0.8 |
| **Age, years** (>50 vs ≤50) | 0.7 (0.4, 1.0); p=0.07 | 0.7 (0.4, 1.1); p=0.1 | 0.7 (0.4, 1.1); p=0.1 |
| **Disease Duration, years** (>10 vs ≤10) | 0.3 (0.2, 0.6); p=0.001 | 0.3 (0.2, 0.6); p=0.001 | 0.3 (0.2, 0.6); p=0.001 |
| **RF Positive** (vs negative) | 1.0 (0.6, 1.5); p=0.9 | 1.0 (0.6, 1.6); p=1.0 | 1.0 (0.6, 1.6); p=0.9 |
| **Erosions on X-ray** (vs negative) | 1.1 (0.7, 1.7); p=0.7 | 1.5 (0.9, 2.4); p=0.1 | 1.5 (0.9, 2.5); p=0.09 |
| **Methotrexate at registration** (vs none) | 0.9 (0.6, 1.5); p=0.8 | 0.9 (0.6, 1.4); p=0.6 | 0.9 (0.6, 1.4); p=0.7 |
| **On steroids at registration** (vs none) | 1.3 (0.8, 2.0); p=0.3 | 1.3 (0.8, 2.1); p=0.3 | 1.3 (0.8, 2.1); p=0.3 |
| **Tender Joint Count** (per joint) | 1.0 (1.0, 1.0); p=0.3 | 1.0 (0.9, 1.1); p=0.7 | 1.0 (0.9, 1.1); p=0.9 |
| **Swollen Joint Count** (per joint) | 1.0 (1.0, 1.0); p=0.2 | 1.0 (1.0, 1.1); p=0.4 | 1.0 (1.0, 1.1); p=0.4 |
| **Patient Global Assessment** (cms) | 1.1 (1.0, 1.2); p=0.1 | 1.1 (0.9, 1.2); p=0.5 | 1.0 (0.9, 1.2); p=0.6 |
| **ESR** (mm/hr) | 1.0 (1.0, 1.0); p=0.2 | 1.0 (1.0, 1.0); p=0.4 | 1.0 (1.0, 1.0); p=0.5 |
| **DAS28** (whole unit) | 1.1 (0.9, 1.3); p=0.4 | 1.1 (0.6, 2.2); p=0.8 | 1.1 (0.5, 2.1); p=0.8 |
| **HAQ** (whole unit) | 1.4 (1.0, 1.9); p=0.05 | 1.4 (1.0, 2.0); p=0.065 | 1.5 (1.0, 2.1); p=0.04 |
| **Total Comorbidities^#^** (vs none) |  |  |  |
| 1 comorbidity | 1.3 (0.8, 2.0); p=0.3 | 1.2 (0.7, 1.9); p=0.5 | 1.1 (0.7, 1.9); p=0.6 |
| 2 comorbidities | 1.1 (0.6, 2.1); p=0.7 | 1.0 (0.5, 2.0); p=1.0 | 1.0 (0.5, 1.9); p=1.0 |
| 3+ comorbidities | 0.8 (0.3, 2.3); p=0.7 | 0.7 (0.3, 2.0); p=0.6 | 0.7 (0.2, 2.1); p=0.5 |
| **Smoke Status** (vs never smoked) |  |  |  |
| Current Smoker | 1.4 (0.8, 2.3); p=0.3 | 1.2 (0.7, 2.1); p=0.5 | 1.3 (0.7, 2.3); p=0.4 |
| Ex-Smoker | 1.1 (0.7, 1.7); p=0.8 | 1.0 (0.6, 1.7); p=0.9 | 1.1 (0.6, 1.8); p=0.8 |
| **Obese (body mass index ≥ 30)** | 1.7 (1.1, 2.6); p=0.03 | 1.6 (1.0, 2.5); p=0.07 | 1.6 (1.0, 2.6); p=0.06 |
| **SF-36: Physical Component Score** | 1.0 (1.0, 1.0); p=0.2 | - | - |
| **SF-36: Mental Component Score** | 1.0 (1.0, 1.0); p=0.5 | 1.0 (1.0, 1.0); p=0.7 | 1.0 (1.0, 1.0); p=0.7 |
| **IMD (Excluding Northern Ireland)**  (all other patients as referent) |  |  |  |
| Lowest Quintile [more deprived] | 1.1 (0.7, 1.8); p=0.8 | - | 0.9 (0.6, 1.6); p=0.8 |

*Results are presented as hazard ratios (HR) with 95% confidence intervals (CI). ^#^Total comorbidities = hypertension, ischemic heart disease, stroke, lung disease, renal disease, diabetes, depression, liver disease. ^##^Patients with IMD data, excluding Northern Ireland (N=1801). Biologic disease modifying anti-rheumatic drugs (bDMARD), Tumour necrosis factor-alpha inhibitor (TNFi), rheumatoid factor (RF), erythrocyte sedimentation rate (ESR), 28-joint disease activity score (DAS28) [higher score indicates worse health], health assessment questionnaire (HAQ) [higher score indicates worse health], 36-item short form survey for quality of life (SF-36) [higher score indicates improved health], index of multiple deprivation (IMD).*
